# Increased RhoA Prenylation in the *loechrig (loe)* Mutant Leads to Progressive Neurodegeneration

**DOI:** 10.1371/journal.pone.0044440

**Published:** 2012-09-06

**Authors:** Mandy Cook, Priya Mani, Jill S. Wentzell, Doris Kretzschmar

**Affiliations:** Center for Research on Occupational and Environmental Toxicology, Oregon Health & Sciences University, Portland, Oregon, United States of America; Virginia Commonwealth University, United States of America

## Abstract

The *Drosophila* mutant *loechrig (loe)* shows age-dependent degeneration of the nervous system and is caused by the loss of a neuronal isoform of the AMP-activated protein kinase (AMPK) γ-subunit (also known as SNF4Aγ). The trimeric AMPK complex is activated by low energy levels and metabolic insults and regulates multiple important signal pathways that control cell metabolism. A well-known downstream target of AMPK is hydroxyl-methylglutaryl-CoA reductase (HMGR), a key enzyme in isoprenoid synthesis, and we have previously shown that HMGR genetically interacts with *loe* and affects the severity of the degenerative phenotype. Prenylation of proteins like small G-proteins is an important posttranslational modification providing lipid moieties that allow the association of these proteins with membranes, thereby facilitating their subsequent activation. Rho proteins have been extensively studied in neuronal outgrowth, however, much less is known about their function in neuronal maintenance. Here we show that the *loe* mutation interferes with isoprenoid synthesis, leading to increased prenylation of the small GTPase Rho1, the fly orthologue of vertebrate RhoA. We also demonstrate that increased prenylation and Rho1 activity causes neurodegeneration and aggravates the behavioral and degenerative phenotypes of *loe.* Because we cannot detect defects in the development of the central nervous system in *loe*, this suggests that *loe* only interferes with the function of the RhoA pathway in maintaining neuronal integrity during adulthood. In addition, our results show that alterations in isoprenoids can result in progressive neurodegeneration, supporting findings in vertebrates that prenylation may play a role in neurodegenerative diseases like Alzheimer’s Disease.

## Introduction

The *loechrig (loe)* mutant was isolated in a screen for mutants that show progressive neurodegeneration of the adult central nervous system. Whereas the brains of newly eclosed *loe* flies show no obvious defects, they begin to display spongiform lesions within all brain regions after a few days of adult life [1]. These lesions increase with further aging and are accompanied by neuronal cell death, ultimately leading to the early death of *loe* flies after about three to four weeks. The *loe* gene encodes the γ-subunit of AMP-activated protein kinase (AMPK), a complex consisting of the catalytic α-subunit and the two regulatory subunits β and γ. The latter contains the binding sites for AMP thereby regulating the activation of the complex [2], [3]. The mutation in *loe* is caused by the insertion of a P-element and affects only one alternative splice form that is strongly expressed in the nervous system [1]. The protein isoform encoded by this transcript contains a unique N-terminus not shared by any of the other isoforms and only this isoform can rescue the *loe* phenotype when expressed in neurons, confirming the requirement of this specific isoform for the integrity of the nervous system [1]. AMPK has been primarily studied as a regulator of energy homeostasis but recently it has been implicated in a plethora of other cellular functions including maintenance of cell polarity and insulin signaling [4]. Whereas vertebrates have several genes for each subunit, flies contain only a single gene for each subunit, greatly facilitating a genetic analysis of AMPK’s function in *Drosophila*. Mutations in the fly α-subunit are lethal and show defects in mitosis and cell polarity as well as stress-induced abnormalities in the actin cytoskeleton [5], [6]. *alicorn* mutants, which affect the β-subunit are also lethal but clonal analyses in the adult revealed a progressive degeneration [7] which together with the *loe* mutation suggests a role of AMPK in neuronal survival. Although the data are sparse and contradictory, a neuroprotective role of AMPK in vertebrates was suggested by the activation of AMPK in several models of pathological stress situations like cerebral ischemia and stroke [8]. In addition, a mutation in PRKAG2, the γ2 isoform of human AMPK, can lead to Wolff-Parkinson-White syndrome, which causes hypertrophic cardiomyopathy and arrhythmia [9].

One of the well-known targets of AMPK is hydroxymethylglutaryl-CoA reductase (HMGR), the rate-limiting enzyme in cholesterol synthesis [2]. Although the function of AMPK as a negative regulator of HMGR is conserved in *Drosophila* [1], this pathway is not involved in cholesterol synthesis in flies because they lack several enzymes required for the de novo synthesis of cholesterol [10]. However, HMGR also regulates isoprenoid synthesis, a pathway that is conserved in insects [10]. Mevalonate, the product of HMGR, can be converted into farnesyl-diphosphate and geranylgeranyl-diphosphate which provide, among other functions, covalently bound lipid moieties for the isoprenylation of membrane-targeted proteins [11]. One class of proteins, known to be regulated by prenylation are GTPases like Ras, Rab, Rac, and Rho, resulting in their association with membranes and subsequent activation [12]. The Rho family of GTPases comprises a major branch of the Ras superfamily and like Ras, Rho proteins function as GTP/GDP switches and alternate between an active GTP-bound state and an inactive GDP-bound state [13]. Due to their role in actin polymerization, Rho-GTPases affect many functions of the cellular cytoskeleton depending on the cell type. Although changes in the actin cytoskeleton have been connected with neuronal survival and neurodegenerative diseases much less is known about the role of prenylation in this process. Promoting prenylation by adding geranylgeranyl-diphosphate (GGPP) resulted in increased levels of Aβ peptides [14] which accumulate in the plaques characteristic for Alzheimer’s Disease. In addition, it was shown that statins, known inhibitors of HMGR, can affect Aβ production by facilitating the non-amyloidogenic β-processing instead of the alternative β-processing that leads to Aβ production [15], [16]. Statin treatment has also been used to investigate the role of prenylation in neurite outgrowth however the results have been inconsistent. Whereas some groups have reported that statin treatment promotes neurite outgrowth, others describe an inhibiting effect [17]. At least in part, these controversial results might be due to the use of different cell lines and/or statins. In flies, statin treatment has been shown to decrease isoprenylated Ras and Rab4, accompanied by an increase in lifespan and an improvement in cardiac health [18].

Here we show that the *loe* mutation affects the regulatory function of AMPK on isoprenoid synthesis, leading to increased RhoA prenylation and progressive neurodegeneration.

## Results

### Interfering with Isoprenoid Synthesis Affects the Neurodegenerative Phenotype of *loe*

**Figure 1:**
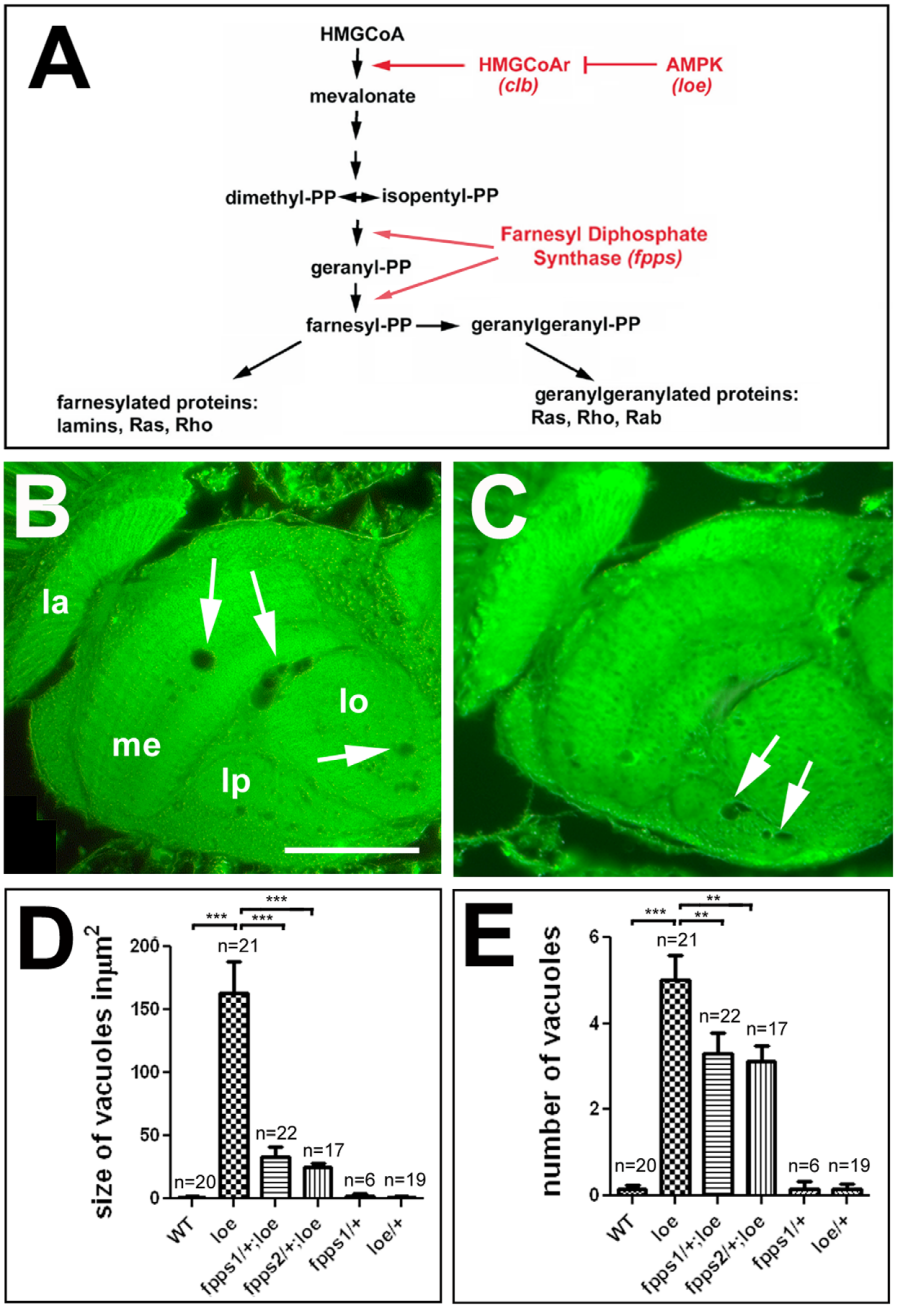
*loe* interferes with the isoprenoid pathway. A. Schematic of the isoprenoid synthesis pathway. **B.** Paraffin head section of a 5d old homozygous female *loe* fly reveals several vacuoles (arrows). **C.** Section of an age-matched female *fpps^k03514^*/+;*loe/loe* fly shows less and smaller vacuoles (arrows). **D, E.** Measuring the area (**D**) and number (**E**) of vacuoles shows that *loe* homozygous flies which are also heterozygous mutant for the farnesyl diphosphate synthase *(fpps)* allele *fpps^k03514^* or *fpps^k06103^* show less vacuolization than *loe* alone. Heterozygous *fpps^k03514^* or heterozygous *loe* flies are indistinguishable from wild type. Flies were 5 d old females. SEMs are indicated, n = number of brain hemispheres analyzed; ** p<0.01, *** p<0.001. la = lamina, me = medulla, lo = lobula, lb = lobula plate. Scale bar in **B** = 50 µm.

As previously shown, flies homozygous for *loe* and heterozygous for lethal alleles of HMGR, which in *Drosophila* is encoded by the *columbus* gene [19], show a suppression of the degenerative phenotype compared to *loe* alone [1]. These experiments confirmed that the inhibitory function of AMPK on HMGR is conserved in flies and that changes in the activity of HMGR play a role in the observed degenerative phenotype. HMGR is a key factor in cholesterol synthesis but also in isoprenoid synthesis, a pathway conserved in *Drosophila* (Fig. 1A). To specifically interfere with the isoprenoid pathway, we crossed *loe* with flies carrying mutations in the farnesyl diphosphase synthase *(fpps)* gene. FPPS is catalyzing the steps to produce geranyl-pyrophosphate and farnesyl-pyrophosphate that are subsequently used to provide the farnesyl and geranylgeranyl moieties attached to target proteins. For this experiment, we compared *loe* flies and *loe* flies that also carried a mutation in *fpps*. The *loe* control flies were obtained from the same cross but carried the CyO balancer instead of the *fpps* mutations to minimize genetic background effects. Analyzing head sections from five day old female flies homozygous for *loe* (Fig. 1B) with age-matched flies that in addition carry one mutant copy of the *fpps^k03514^* (Fig. 1C) or *fpps^k06103^* allele [20] revealed a significant suppression of the degenerative phenotype. Measuring the area of vacuoles in these flies in the optic system, we found a reduction from 163±24.5 µm^2^ in *loe* alone to 33±8.9 µm^2^ and 25±3.4 µm^2^ in *loe* carrying one copy of *fpps^k03514^* or *fpps^k06103^* (Fig. 1D; p<0.001). Similarly, *loe* flies showed a reduction in the number of vacuoles from 5±0.6 to 3.3±0.5 and 3.1±0.4 when also one copy of *fpps* was mutant, confirming an involvement of the isoprenoid pathway in the degenerative phenotype of *loe*. Wild type flies, heterozygous *fpps^k03514^* mutants, or heterozygous *loe* mutants showed no degenerative phenotype with only occasionally a small vacuole forming (Fig. 1D and 1E). Due to the lethality of homozygous mutant *fpps* flies during development these could not be analyzed as adults.

**Figure 2:**
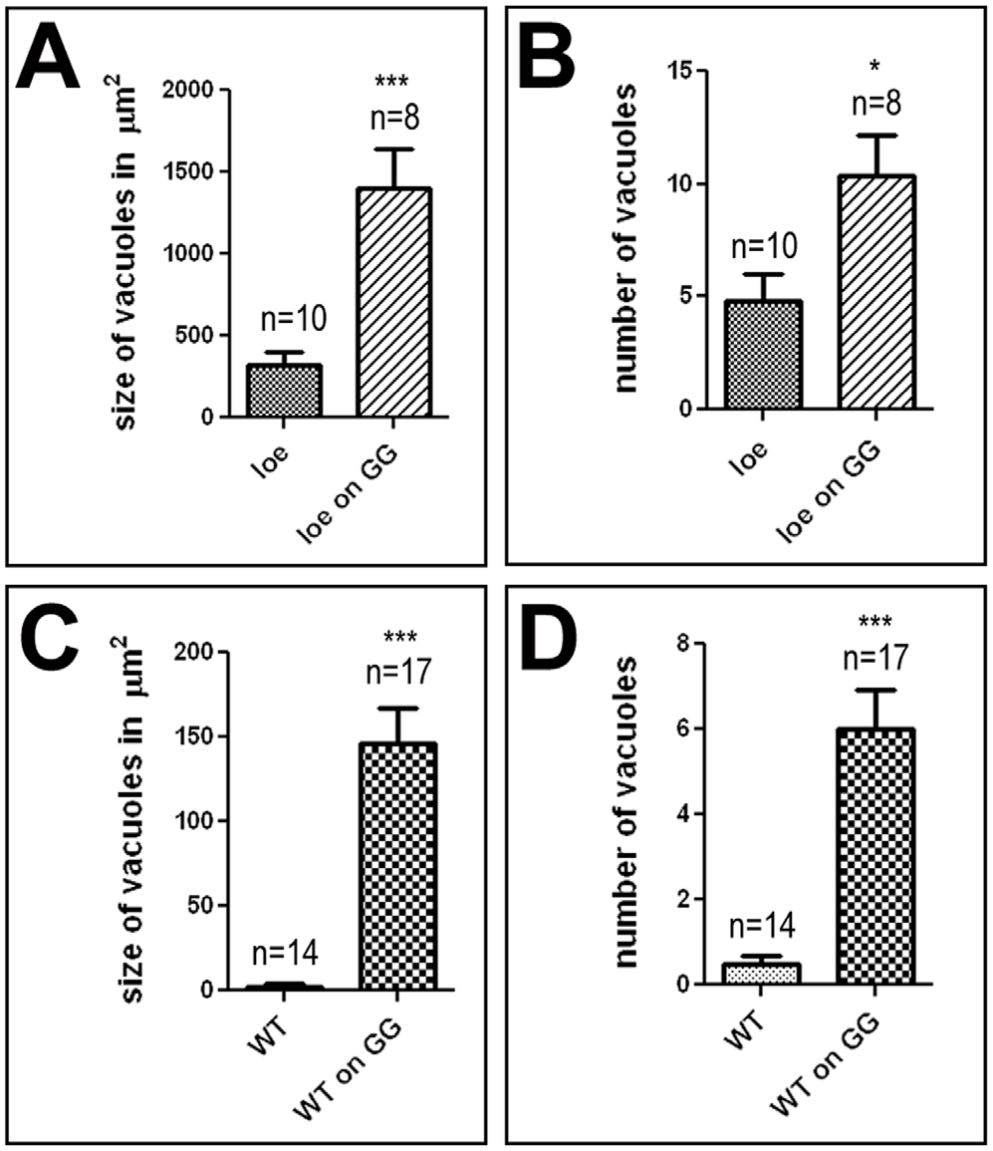
Feeding geranyl geraniol aggravates or induces degeneration. **A, B.** Feeding *loe* fies 10 mM geranyl geraniol (GG) in 5% glucose significantly increases the size of vacuoles (**A**) as well as their number (**B**). **C, D.** Geranyl geraniol treatment also induced vacuole formation in wild type flies. All flies were 7 d old females. SEMs are indicated, n = number of brain hemispheres analyzed; * p<0.05, *** p<0.001.

In addition to these genetic experiments, we performed pharmacological manipulations using farnesol and geranyl-geraniol to increase isoprenylation. Whereas we did not detect an effect of farnesol, feeding geranyl-geraniol in 5% glucose enhanced the degeneration in seven day old *loe* flies from 317±89 µm^2^ to 1402±233 µm^2^ (p<0.001; Fig. 2A). Similarly, the number of vacuoles was increased from 4.8±1.2 to 10.4±1.7 in the treated *loe* flies (p<0.05; Fig. 2B) further supporting a role of altered isoprenoid levels in the degeneration observed in *loe*. In addition, we tested whether feeding geranyl-geraniol can induce degeneration in wild type flies and as shown in Figure 2C, we found significant vacuolization in wild type flies treated for seven days with 10 mM geranyl-geraniol compared to untreated controls. Whereas control showed only a few (0.5±0.2), very small vacuoles (3±1.133 µm^2^), treated wild type flies had an average of 6±0.9 vacuoles with a total area of 146±21.2 µm^2^ (both p values <0.0001). These results strongly suggest that an increase in isoprenoid production can lead to a neurodegenerative phenotype.

### Decreasing Rho1 Suppresses *loe*-induced Phenotypes

**Figure 3:**
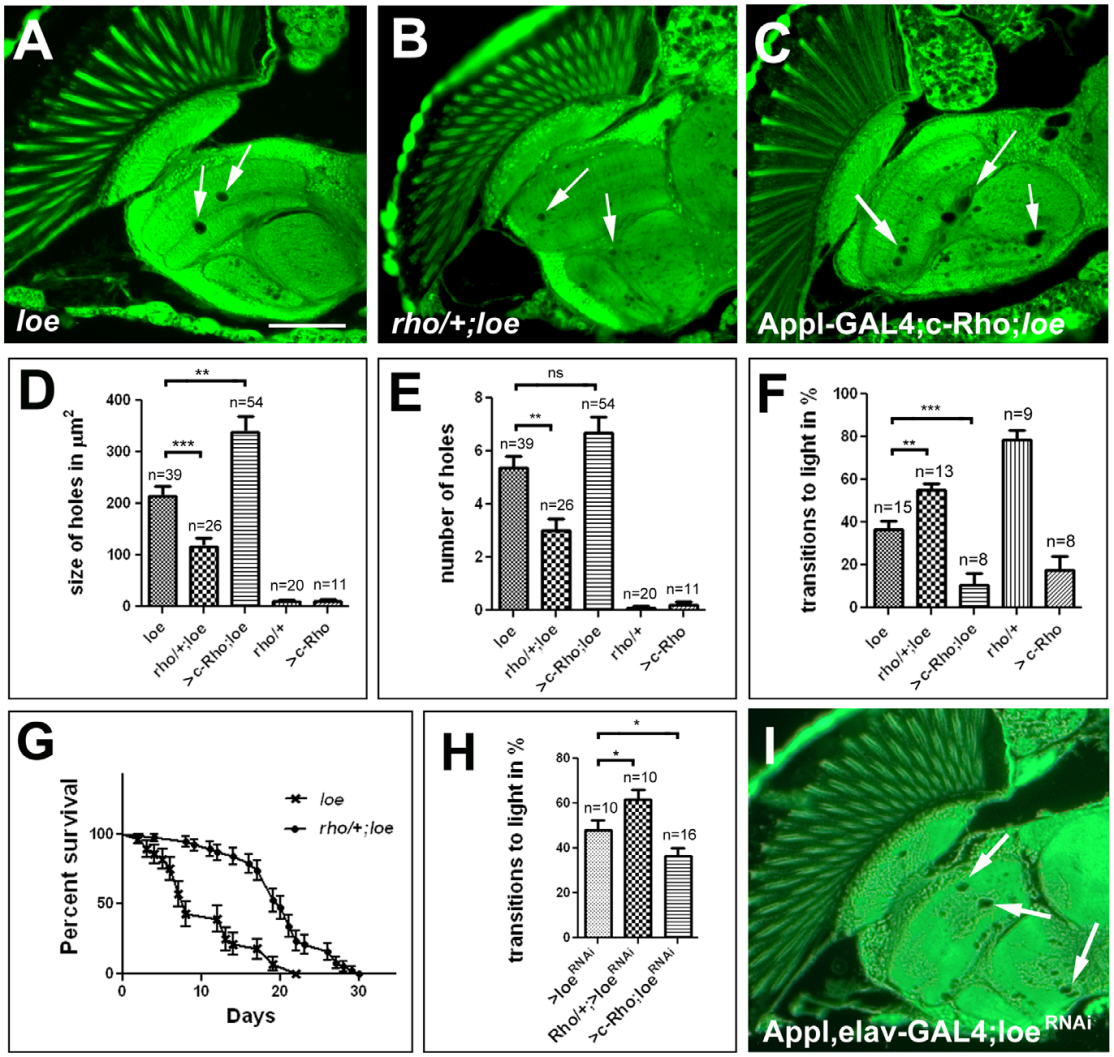
*loe* interacts with Rho1. **A.** A section from a *loe* control fly shows more vacuolization (arrows) than a *Rho^72F^*/+; *loe/loe* fly (**B**). **C.** In contrast, a fly expressing constitutive active Rho1 in the nervous system using *Appl*-GAL4 exhibits more spongiform lesions than the *loe* control (**A**). **D, E.** Quantification of the vacuolization reveals a significant reduction in the size (**D**) and number (**E**) of vacuoles in *Rho^72F^*/+; *loe/loe* flies compared to controls obtained from the same cross whereas flies expressing constitutive active Rho^V14^ show an enhancement. *Rho^72F^*/+ flies or *Appl*-GAL4>Rho1^V14^ flies alone do not show a degenerative phenotype. n = analyzed brain hemispheres. **F.** Similarly, *Rho^72F^*/+; *loe/loe* flies performed better in the fast phototaxis assay than *loe* alone whereas expressing Rho1^V14^ via *Appl*-GAL4 reduced their performance. n = number of independent experiments with groups of 5–10 flies (groups of 3–6 were tested when Rho1^V14^ was expressed because only few of these flies survived). **A–F** All flies were 5 d old females. **G.** Heterozygosity for *Rho^72F^* also significantly increased the lifespan in *loe* females (p<0.001). **H.** Inducing a loe^RNAi^ construct with *elav*-GAL4 also resulted in deficits in the phototaxis assay that are partially suppressed when combined with *Rho^72F^*/+ and enhanced when Rho1^V14^ is co-expressed. n = number of groups of 5–10 females 14 d old. **I.** A 14 d old male fly in which the loe^RNAi^ construct was induced with *elav*-GAL4 and *Appl*-GAL4 shows vacuole formation (arrows) similar to *loe* mutant flies. SEMs are indicated in all the graphs. * p<0.05, ** p<0.01, *** p<0.001. Scale bar in **A** = 50 µm.

As pointed out above, small GTPases are well known targets of isoprenylation and we therefore investigated whether *loe* genetically interacts with mutations in Rho-GTPases. Whereas we could not detect any significant effects of removing one copy of cdc42 or Rab5 in *loe* (215±17 µm^2^ and 174±22 µm^2^ versus 182±29 µm^2^) heterozygosity for Rho1, the fly orthologue of vertebrate RhoA, resulted in a suppression of the vacuolization. Combining one copy of *Rho^72F^*, an allele that deletes part of the coding region including the translation start site [21], with *loe* (Fig. 3B) reduced the vacuolization almost by half in 5 d old flies with 117±16 µm^2^ compared to 208±21 µm^2^ in control *loe* flies (Fig. 3A, 3D; p<0.001). In addition, we used a constitutively active form of Rho1 (Rho^V14^; [22] to determine whether increased Rho activity can aggravate the degeneration in *loe*. As shown in Figure 3C and 3D, expression of this construct in the nervous system, using a pan-neuronal *Appl*-GAL4 promoter line enhanced the degeneration to 338±29 µm^2^ (p<0.01). Neither heterozygous *Rho^72F^* flies, nor *Appl*-GAL4;UAS-Rho^V14^ flies showed significant vacuolization at this age (9.5±2.7 and 11.0±3.3 µm^2^, respectively). Counting the number of vacuoles in these flies (Fig. 3E) also revealed a significant reduction when *loe* is heterozygous mutant for *Rho^72F^* with 5.4±0.4 compared to 3.0±0.5 (p<0.01) but although the number was increased in *loe* flies expressing the constitutive active Rho, the difference was not quite statistically significant (6.7±0.6; p = 0.08).

To complement these data, we also investigated whether Rho levels affect the behavioral phenotype and lifespan of *loe*. Indeed, heterozygosity for *Rho^72F^* increased the performance index of *loe* flies in the fast phototaxis assay (36.7±4.1 to 55.1±3.2, Fig. 3F) whereas expression of Rho^V14^ via *Appl*-GAL4 decreased it significantly (to 10.6±5.2). Heterozygous *Rho^72F^* flies alone performed quite well however, *Appl*-GAL4;UAS-Rho^V14^ alone performed even worse than *loe* with 17.8±6.3 although these flies do not reveal significant vacuolization at this age (see Fig. 3E and 3F). *Rho^72F^*/+; *loe/loe* flies also showed a significant increase in lifespan compared to *loe* alone (p<0.001, Fig. 3G), confirming that heterozygosity for Rho also suppresses other deleterious phenotypes of *loe*. To finally verify a genetic interaction of Rho1 and *loe*, we used an RNAi construct that targets one of the common exons of *loe*. Inducing this construct pan-neuronally via *elav*-GAL4 did not result in a detectable degenerative phenotype in 14 d old flies (data not shown), however the flies revealed only a performance index of 48±4.1 in the fast phototaxis assay (Fig. 3H). Using this phenotype for a genetic interaction test also showed a suppression when Rho1 levels were reduced by heterozygosity for *Rho^72F^* and enhanced when constitutive active Rho1 was co-expressed (61.5±4.7 and 36.5±3.6; Fig. 3H). Finally, we tested whether we can induce a degenerative phenotype by the knockdown when increasing the number of drivers or by co-expressing dicer. As shown in Figure 3I, inducing the RNAi construct with *elav*-GAL4 and *Appl*-GAL4 did result in a degenerative phenotype in 14 d old flies similar to young *loe* mutants. A similar result was obtained by co-expression of dicer and the RNAi construct with *Appl*-GAL4 data not shown).

### *loe* Mutants have Decreased Levels of Total Rho1 but Increased Levels of Prenylated Rho1

**Figure 4:**
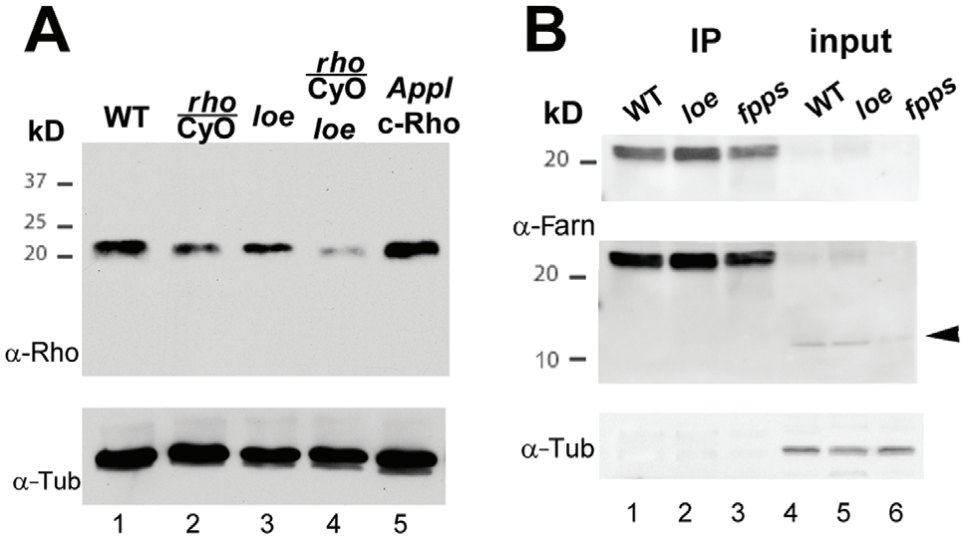
*loe* affects the levels and isoprenylation of Rho1. **A.** Western Blot showing reduced levels of total Rho1 protein in *loe* flies (compare lane 1 and 3 or lane 2 and 4). As expected, removing one copy of the *Rho1* gene decreases the levels of Rho1 (compare lane 1 and 2) whereas expression of the constitutively active Rho1 increases the amount of total Rho1 (compare lane 1 and 5). **B.** Immunoprecipitations from head lysates using anti-Rho1 probed with an anti-farnesyl antibody (upper panels) show an increase in prenylated Rho1 in *loe* (lane 2). In contrast, prenylated Rho1 is decreased in heterozygous *fpps^k03514^* flies (lane3), as are other farnesylated proteins that are detectable in the input (arrowhead). Loading controls are shown below.

To confirm that our genetic manipulations did indeed affect the levels of Rho1, we performed Western Blots using an anti-Rho1 antibody. As expected, flies heterozygous for *Rho^72F^* showed a significant reduction in the levels of Rho1 compared to wild type flies (Fig. 4A, lane 1 and 2) whereas flies expressing constitutive active Rho1 (Rho1^V14^) in the nervous system revealed increased levels (*Appl*; c-Rho; lane 5). Surprisingly, we also found that *loe* flies showed a decrease in the levels of Rho1 (lane 3 and 4), suggesting that a compensatory mechanism exists that reduces the total levels of Rho1 when too much active Rho1 is present.

**Figure 5:**
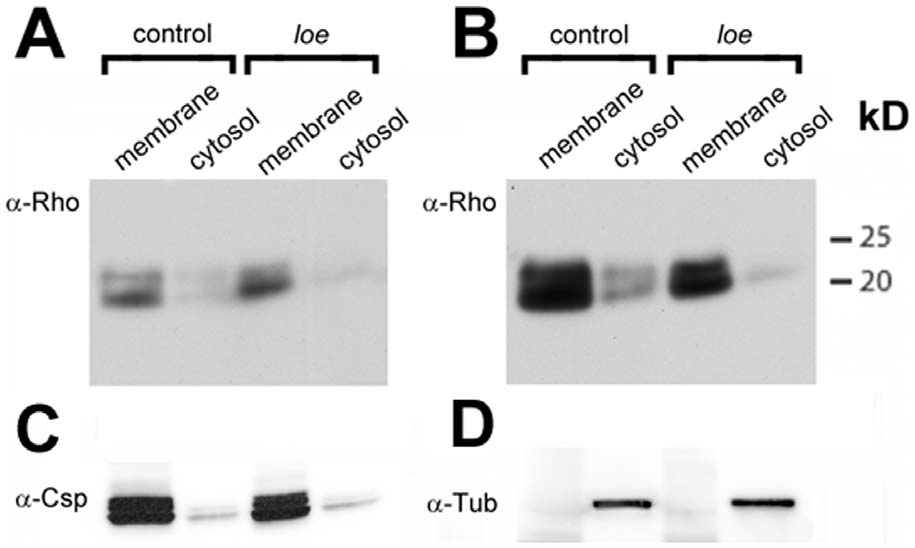
*loe* affects the intracellular localization of Rho1. **A.** A Western Blot using membrane and cytosolic fractions shows an increase of membrane-associated Rho1 in *loe*. **B.** In contrast, in the cytosolic fraction of *loe* the levels of Rho1 are decreased compared to the control (shown is the same Western Blot as in (**A**) but with a longer exposure time). 4 µg of total protein were loaded in each lane. **C, D.** Antibodies against the synaptic vesicle-associated protein CSP and the cytosolic Tubulin are used as loading controls and as a marker for the efficiency of the fractionation.

Although the effect of *loe* on the total amount of Rho1 protein was unexpected, we did expect that *loe* would affect the levels of prenylated Rho1. To confirm this, we immunoprecipitated Rho1 from head lysates using an anti-Rho1 antibody followed by Western Blots with an anti-farnesyl/geranylgeranyl antibody. As shown in Figure 4B, *loe* flies show increased levels of prenylated Rho1 (lane 2) whereas they are decreased in *fpps^k03514^* heterozygous mutants (lane 3). That *fpps^k03514^* mutants generally decrease the levels of isoprenylation is seen in the longer exposure in the input lane (lane 6) by the reduction of an unknown anti-farnesyl positive band (arrowhead, Gig. 4B). In contrast, this band appears not affected by *loe* (lane 5), suggesting that *loe* does not affect all prenylated proteins. Because the attachment of isoprenyl moieties facilitates membrane association of small GTPases, we also analyzed the membrane localization of Rho1 in wild type and *loe* flies. Comparing membrane and cytosolic fractions in Western Blots, we did indeed detect a slight increase in membrane associated Rho1 in *loe* (Fig. 5A, upper panel) together with a reduction in the levels of cytosolic Rho1 (Fig. 5B, upper panel). The efficiency of the fractionation of membranes and cytosol was confirmed by staining with an antibody against the membrane associated Cystein String Protein (Fig. 5A, lower panel) and the cytosolic Tubulin (Fig. 5B, lower panel). These experiments verify that *loe* mutants have more prenylated, membrane-associated Rho1.

### Overexpression of Activated Rho1 Induces a Neurodegenerative Phenotype

**Figure 6:**
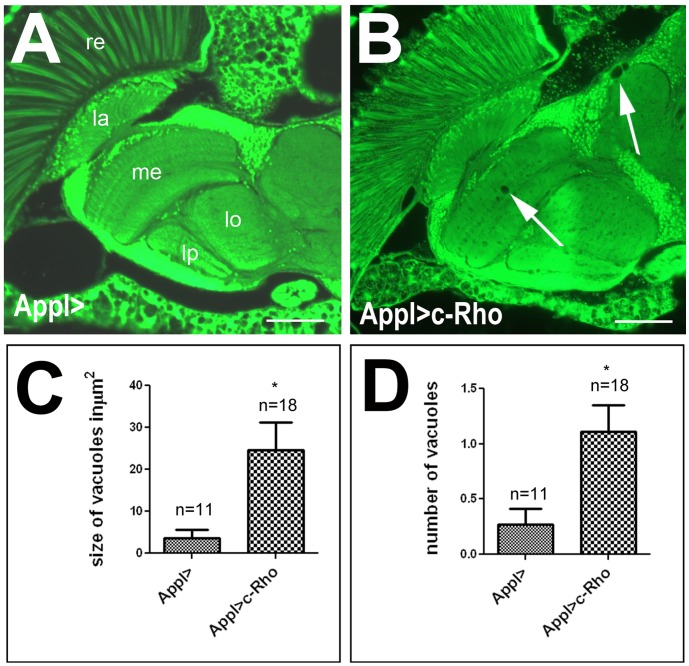
Neuronal expression of Rho1^V14^ results in neurodegeneration. **A.** A 14d old control fly only containing the *Appl*-GAL4 construct does not show signs of degeneration. **B.** Inducing the constitutive active Rho1^V14^ construct pan-neuronally using *Appl*-GAL4 results in the formation of a few vacuoles in the brain (arrows) of a 14 d old fly. **C, D.** Quantification of the area of vacuoles (**C**) and number of vacuoles (**D**) confirms a significant increase in degeneration in these flies. The number of analyzed brain hemispheres and the SEM is indicated. *<0.05. re = retina, la = lamina, me = medulla, lo = lobula, lb = lobula plate. Scale bar = 30 µm.

Next we investigated whether a rise in active Rho1 can cause neurodegeneration in a wild type background. For this purpose, we expressed the constitutively active form pan-neuronally using *Appl*-GAL4. Although we did not detect vacuoles in young flies (5 d, see Fig. 3D, 3E), a few vacuoles were detectable in 14d old flies (arrows, Fig. 6B) in contrast to age-matched controls only carrying *Appl*-GAL4 which did not show any vacuoles (Fig. 6A). A quantification confirmed that the total size of vacuoles (24.6±6.7 µm^2^ versus 3.6±1.9 µm^2^; Fig. 6C) as well as their number (0.3±0.1 versus 1.1±0.2; Fig. 6D) was significantly increased in the Rho1^V14^ expressing flies compared to the controls.

**Figure 7:**
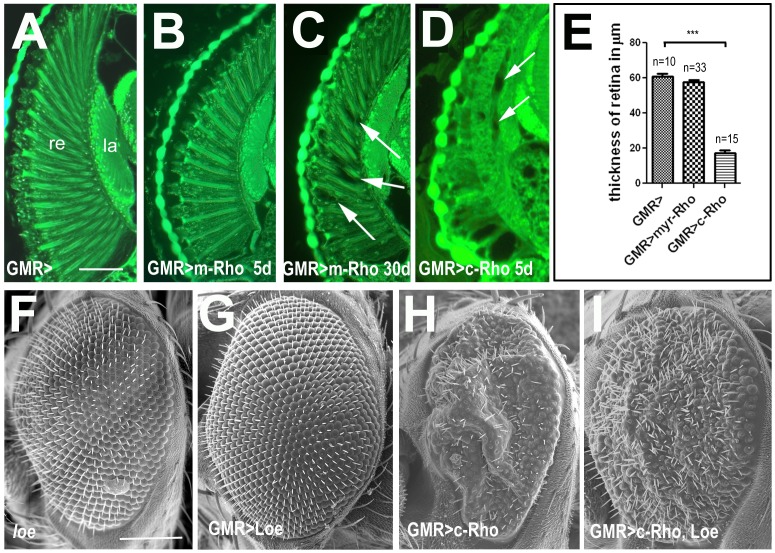
Increased activation of Rho1 in the eye leads to retinal degeneration. **A.** A 30 d old control fly carrying only the GMR-GAL4 construct reveals an intact retina. **B.** The retina is also still intact in a 5 d old fly expressing the myristilated Rho1 in the eye via GMR-GAL4. **C.** In contrast, vacuoles (arrows) have formed in the retina in GMR-GAL4; UAS-myr-Rho1flies when aged for 30 d. **D.** Expressing the constitutive active Rho1^V14^ via GMR-GAL4 results in a more severe degeneration with vacuoles detectable in the retina and lamina (arrows) of a 5 d old fly. **E.** Measuring the thickness of the retina in 14 d old flies reveals a significant reduction in size in flies expressing the constitutive active Rho1^V14^ via GMR-GAL4 whereas expression of the myristilated Rho1 did not affect retinal thickness. The number of measured retinae and the SEM is indicated; ***<0.001. **F–I.** External eye phenotype in 5 d old flies. **F.** *loe* mutant flies show a slight roughness of the eye, mostly detectable by the disorganized bristle pattern. **G.** Flies overexpressing LOE via GMR-GAL4 do not reveal a phenotype. **H.** In contrast, inducing Rho1^V14^ with GMR-GAL4 results in a smaller and severely disrupted retina, a phenotype that is suppressed by co-expression of UAS-loe (**I**). re = retina, la = lamina. Scale bar in **A** = 30 µm, in **F** = 100 µm.

Inducing the constitutive active Rho1^V14^ in the eye via GMR-GAL4 resulted in a much more dramatic degeneration already in young flies (Fig. 7D; 5 d old), probably due to the high expression levels of this promoter construct. As shown in Figure 7H, this phenotype already affects the development of the retina, causing a smaller and severely rough eye. To determine whether an increase in membrane association was sufficient to induce neurodegeneration, we created a Rho1 construct that carried an N-terminal myristyl group to promote its association with membranes. Expressing this construct in the nervous system using *Appl*-GAL4 did not result in a degenerative phenotype (data not shown), even when aged for 20 d. However, expressing it with the GMR-GAL4 driver in the eye resulted in a progressive degeneration of the retina. Whereas 5 d old GMR-GAL4/UAS-myr-Rho1 flies (Fig. 7B) were indistinguishable from control flies only expressing GMR-GAL4 (Fig. 7A) or only UAS-myr-Rho1 (data not shown), 30 day old GMR-GAL4/UAS-myr-Rho1 flies revealed several vacuoles in the retina (Fig. 7C, arrows). However, this did not affect the thickness of the retina, in contrast to the expression of the constitutive active Rho which resulted in a dramatic decrease of retinal thickness (17.2±1.6 µm compared to 60.8±1.5 µm in controls; Fig. 7E). Nevertheless the vacuole formation in myr-Rho1 expressing flies shows that facilitating the association of Rho1 with membranes is sufficient to induce degenerative effects.

To confirm that the Rho1 induced degeneration can is connected to *loe*, we performed genetic interaction tests using the rough eye phenotype induced by expression of Rho1^V14^ with GMR-GAL4. Though *loe* flies alone have a slightly rough eye (Fig. 7A), *loe* had no detectable effect on the external or internal phenotype caused by Rho1^V14^ (data not shown). However, overexpression of LOE, which by itself has a normal looking eye (Fig. 7G), suppressed the degeneration caused by Rho1^V14^ (compare Fig. 7H and 7I). This suggests that decreased isoprenylation of Rho1^V14^, by inhibition of the isoprenoid synthesis pathway through LOE, reduces its membrane association thereby affecting its interaction with its partners.

## Discussion

Since its first mention in 1973 [23], AMPK has developed into an key regulator of multiple biochemical pathways [24]. In its role as a “stress sensor”, AMPK inhibits such energy demanding processes like cholesterol synthesis by inhibiting hydroxymethylglutaryl (HMG)-CoA reductase, the rate-limiting enzyme in cholesterol synthesis [2]. Though the function of AMPK as a negative regulator of HMGR and most of the downstream biosynthetic pathway from mevalonate is conserved in flies [1], they cannot synthesis cholesterol *de novo*. This is due to the fact that they miss several of the downstream enzymes required for the synthesis of cholesterol from farnesyl pyrophosphate [10]. However, flies have all the enzymes required to produce farnesyl pyrophosphate and geranylgeranyl pyrophosphate. Although, prenylation provides an important mechanism to regulate proteins, including neuronal proteins, the effects of changes in prenylation on neuronal outgrowth, function, and maintenance have not been well studied. Studies that have addressed this issue have mostly used statin treatment as a tool to affect isoprenylation and have provided mixed results. Statin treatment can promote or decrease neurite outgrowth depending on the used cell line, cell type, and/or the specific statin used in the experiment [17].

The *loe* mutant now allowed us to investigate how changes in isoprenoid synthesis can affect the nervous system without relying on pharmacological intervention. That the role of AMPK in regulating isoprenoid synthesis is involved in the progressive degeneration observed in *loe* was first confirmed by the genetic interaction with mutations in the farnesyl diphosphate synthase. Decreasing the expression of FPPS, significantly reduced the vacuole formation in *loe* supporting our hypothesis that the loss of functional neuronal AMPK upregulates the synthesis of isoprenoids. That an increase in these isoprenoid intermediates is deleterious for the nervous system was also supported by feeding geranyl geraniol which not only enhanced the degenerative phenotype in *loe* but also induced lesion in wild type flies.

As mentioned above, farnesyl pyrophosphate and geranylgeranyl pyrophosphate provide lipid moieties that are irreversible attached to the C-terminus of proteins, including small GTPases like Rho proteins, allowing their association with membranes [25]. The targeting to membranes is then thought to facilitate their interaction with guanine nucleotide exchange factors leading to their activation [17]. We therefore tested, whether the effects of changes in isoprenylation might be mediated by one of these proteins. As shown in Figure 3, manipulating the levels of Rho1, the fly RhoA, did indeed aggravate or ameliorate the degeneration and behavioral deficits in *loe*. In contrast, mutations in cdc42 or Rab5 did not modify the *loe* phenotype, which together with the result that some proteins did not show increased isoprenylation in *loe* suggests a certain specificity, possibly due to the restricted expression pattern of loeI to neurons. However, the reduction in Rho1 had less of a suppressing effect than the decrease in FPPS (about 50% with Rho compared to about 80% with FPPS), indicating that increased farnesylation of other proteins besides Rho1 also plays a role in the degenerative phenotype of *loe*.

As expected by the inhibitory function of AMPK on isoprenoid synthesis, reducing the levels of Rho1 was beneficial whereas further increasing the amount of active Rho1 by expressing a constitutive active form enhanced the phenotypes. We also confirmed that *loe* flies accumulate isoprenylated Rho1 and show, a slight increase in membrane associated Rho1. Interestingly, we also observed a decrease in the total levels of Rho1 in *loe* mutant flies, indicating that some feed-back mechanism exists that connects the levels of prenylated active Rho1 with Rho1 transcription or degradation. That an increase in membrane association and activation of Rho1 is sufficient to induce neurodegenerative phenotypes was confirmed by the results that expression of myristylated or constitutive active Rho1 also resulted in progressive degeneration, whereby the constitutive active Rho1 had more severe effects. However, in both cases the neurodegneration was not as severe as in *loe*, suggesting that the phenotype in this mutant is due to a pleitropic effect, consistent with the involvement of AMPK in a variety of other pathways.

RhoA has been extensively studied in neuronal development, whereby its activation has generally been connected with growth cone collapse and axon retraction [26], [27]. However, in some instances it can also promote neurite outgrowth [28]. As published previously [1], [29], we could not detect defects in the brains of 1st instar larvae or newly eclosed adult *loe* flies, suggesting that embryonic as well as pupal development of the central nervous system is not affected in this mutant. This is probably due to the fact that the loeI transcript, which is the only one affected by the mutation, is not or only very weakly expressed during development [1]. However, *loe* mutant flies have a slightly rough eye, although this mainly affects the pattern of the bristles and we did not detect effects on the photoreceptors, suggesting that it does play a role in late pupal development of the eye. In contrast, to the weak expression during development, loeI is strongly expressed in the adult head, therefore probably mostly interfering with the posttranscriptional regulation of Rho1 in the adult nervous system. This would also be in agreement with the fact that we did not observe an enhancing effect of the *loe* mutation on the rough eye phenotype caused by the expression of constitutive active Rho1. Alternatively, this phenotype is too severe to detect further enhancement by *loe*. Interestingly, induction of LOE in the eye did suppress this phenotype, presumably by decreasing the isoprenylation of the constitutive active Rho1 thereby interfering with its membrane localization and interaction with its nucleotide exchange factors. In addition to this developmental phenotype, our results also show a requirement of Rho1 for the integrity and survival of established neurons. This is shown be the results that expressing myristylated Rho1 in the eye causes a progressive degeneration and not a developmental defect and also the degenerative phenotype after pan-neuronal expression of the constitutive active Rho1 was only detectable in aged animals. Interestingly, RhoA prenylation and the activation of its downstream target ROCK have been connected with the metabolism of the Amyloid Precursor Protein and Alzheimer’s Disease [30], suggesting that the posttranslational regulation of RhoA by isoprenylation also plays a role for the integrity of the nervous system in vertebrates. In addition, it has been shown that changes in genes that encode proteins involved in prenylation can lead to rare diseases like Hutchinson syndrome, retinitis pigmentosa and cancer [31].

## Materials and Methods

### Fly Stocks

All fly stocks were maintained and raised under standard conditions at 25°C. Canton S was used as wild type control. The *Appl*-GAL4 line was kindly provided by L. Torroya (Universidad Autonoma de Madrid, Spain). The *loe* mutation is described in [1]. *fpps^k03514^, fpps^k06103^* [20], *Rho^72F^* [21], Rho1^V14^.^Scer\UAS^ [22], and the RNAi line SNF4Aγ^JF02060^ were obtained from the Bloomington stock center. UAS-myr-Rho1 flies were created by attaching a myr-tag (kindly provided by M. Wehrli, OHSU) to the coding region of Rho1 and cloning it into pUAST [32] for P-element transformation using the BestGene Transgenic Service.

### Feeding Experiments

After eclosion flies were kept in empty plastic vials deprived of normal food sources and nutrition was provided in the form of 5% glucose solution with or without the compound of interest. A small piece of tissue was soaked in 500 µl glucose solution and added to the vialsin an open Eppendorf tube. Geranyl geraniol and farnesol (both obtained from Sigma) were used at 5 mM and 10 mM.

### Tissue Sections and Measurement of the Vacuolar Pathology

Paraffin sections were performed as described in [33]. To quantify the vacuolization, we photographed sections that showed the entire optic system and the worst phenotype. For a double blind analysis, pictures were taken without knowing the genotype and numbered. The area of the vacuoles in the lamina, medulla, lobula, and lobula plate of each brain hemisphere was then calculated in Photoshop as total pixel number, converted into µm^2^, and the genotype determined [33]. In addition, we counted the number of vacuoles in each picture. Thickness of retinae was measured using ImageJ. As controls for the crosses with *loe*, we used flies from the same cross, which did not carry the UAS construct and flies which had a balancer chromosome instead of the additional mutation to minimize effects by the genetic background. Statistics were done using GraphPad Prism and one-way ANOVA. A Student’s Neuman-Keuls test was used to identify the significantly when only two groups were compared.

### Scanning Electron Microscopy

Heads were prepared and fixed as described in [34], critical point dried and sputtered with a 16 nm layer of gold/palladium. They were then viewed at 10 kV using a FEI Quanta 200 scanning electron microscope.

### Fast Phototaxis and Survival

Fast phototaxis assays were conducted in the dark using the countercurrent apparatus described by [35] and a single light source. A detailed description of the experimental conditions can be found in [36]. Flies were starved overnight, but had access to water and were tested the following morning. Five consecutive tests were performed in each experiment with a time allowance of 6 seconds to make a transition towards the light and into the next vial. A minimum of eight independent tests with groups of 5–10 flies was used per genotype (groups of 3–6 flies were tested for experiment including *Appl*-GAL4; Rho1^V14^ because we did not obtain many of these flies and they also showed an increased lethality during the starving period). For the lifespan analysis, newly eclosed flies were collected and transferred to fresh vials every 4–5 d. ANOVA was applied to data sets to reveal significant differences. Flies were kept in groups of 5–8 flies and at least 5 groups were analyzed. Statistical analysis was done using the analysis for survival curves in GraphPad Prism.

### Western Blots

Heads from 2–3 d old flies were homogenized as described in [1] and proteins separated on 12% SDS-PAGE gels and transferred using the Bio-Rad Mini Trans-Blot® Cell system. Proteins were transferred to Hybond membranes (Amersham Bioscienses). Primary antibodies against Rho1, Cystein String Protein, and Tubulin were obtained from the Developmental Studies Hybridoma Bank developed under the auspices of the NICHD and maintained by the Department of Biology, University of Iowa, and used at 1∶100 in TBST supplemented with 5% nonfat dry milk. The anti-farnesyl/geranyl antiserum was obtained from Abcam and used at 1∶200. Bands were visualized using horseradish peroxidase-conjugated secondary antibodies (Jackson ImmunoResearch) at 1∶1000 and the SuperSignal® West Pico chemiluminiscent substrate (ThermoScientific). At least three replicates were performed.

### Immunoprecipitation and Subcellular Fractionation

For immunoprecipitations approximately 500 fly heads were homogenized and Rho1 precipitated following the protocol in [37], using the anti-Rho antibody and Protein A/G Plus Agarose (Santa Cruz Biotechnology). Membrane and cytosolic fractions were prepared from the different genotypes following the protocol of Orgad et al. [38]. *Appl*-GAL4 flies were used as controls due to their similar eye color to *loe*, minimizing effects caused by varying levels of eye pigment in the Bradford assays. Approximately 300 heads were used for each preparation, protein amounts were determined by Bradford assays [39] and 4 µg total protein loaded per lane.

## References

[1] Tschape JA, Hammerschmied C, Muhlig-Versen M, Athenstaedt K, Daum G, et al.. (2002) The neurodegeneration mutant lochrig interferes with cholesterol homeostasis and Appl processing. Embo J: 21, 6367–6376. 10.1093/emboj/cdf636 PMC136940 12456644

[2] Kemp BE, Mitchelhill KI, Stapleton D, Michell BJ, Chen ZP, et al.. (1999) Dealing with energy demand: the AMP-activated protein kinase. Trends Biochem Sci: 24, 22–25. 10.1016/s0968-0004(98)01340-1 10087918

[3] Hardie DG, Carling D, Carlson M (1998) The AMP-activated/SNF1 protein kinase subfamily: metabolic sensors of the eukaryotic cell? Annu Rev Biochem: 67, 821–855. 10.1146/annurev.biochem.67.1.821 9759505

[4] Hardie DG (2007) AMP-activated/SNF1 protein kinases: conserved guardians of cellular energy. Nat Rev Mol Cell Biol: 8, 774–785. 10.1038/nrm2249 17712357

[5] Lee JH, Koh H, Kim M, Kim Y, Lee SY, et al.. (2007) Energy-dependent regulation of cell structure by AMP-activated protein kinase. Nature: 447, 1017–1020. 10.1038/nature05828 17486097

[6] Mirouse V, Swick LL, Kazgan N, St Johnston D, Brenman JE (2007) LKB1 and AMPK maintain epithelial cell polarity under energetic stress. J Cell Biol: 177, 387–392. 10.1083/jcb.200702053 PMC2064817 17470638

[7] Spasic MR, Callaerts P, Norga KK (2008) Drosophila alicorn is a neuronal maintenance factor protecting against activity-induced retinal degeneration. J Neurosci: 28, 6419–6429. 10.1523/JNEUROSCI.1646-08.2008 PMC6670889 18562613

[8] McCullough LD, Zeng Z, Li H, Landree LE, McFadden J, et al.. (2005) Pharmacological inhibition of AMP-activated protein kinase provides neuroprotection in stroke. J Biol Chem: 280, 20493–20502. 10.1074/jbc.M409985200 15772080

[9] Burwinkel B, Scott JW, Buhrer C, van Landeghem FK, Cox GF, et al.. (2005) Fatal congenital heart glycogenosis caused by a recurrent activating R531Q mutation in the gamma 2-subunit of AMP-activated protein kinase (PRKAG2), not by phosphorylase kinase deficiency. Am J Hum Genet: 76, 1034–1049. 10.1086/430840 PMC1196441 15877279

[10] Santos AC, Lehmann R (2004) Isoprenoids control germ cell migration downstream of HMGCoA reductase. Dev Cell: 6, 283–293. 10.1016/s1534-5807(04)00023-1 14960281

[11] Holstein SA, Hohl RJ (2004) Isoprenoids: remarkable diversity of form and function. Lipids: 39, 293–309. 10.1007/s11745-004-1233-3 15357017

[12] Seabra MC (1998) Membrane association and targeting of prenylated Ras-like GTPases. Cell Signal: 10, 167–172. 10.1016/s0898-6568(97)00120-4 9607139

[13] Boureux A, Vignal E, Faure S, Fort P (2007) Evolution of the Rho family of ras-like GTPases in eukaryotes. Mol Biol Evol: 24, 203–216. 10.1093/molbev/msl145 PMC2665304 17035353

[14] Zhou Y, Su Y, Li B, Liu F, Ryder JW, et al.. (2003) Nonsteroidal anti-inflammatory drugs can lower amyloidogenic Abeta42 by inhibiting Rho. Science: 302, 1215–1217. 10.1126/science.1090154 14615541

[15] Pedrini S, Carter TL, Prendergast G, Petanceska S, Ehrlich ME, et al.. (2005) Modulation of Statin-Activated Shedding of Alzheimer APP Ectodomain by ROCK. PLoS Med: 2, e18. 10.1371/journal.pmed.0020018 PMC543463 15647781

[16] Ostrowski SM, Wilkinson BL, Golde TE, Landreth G (2007) Statins reduce amyloid-beta production through inhibition of protein isoprenylation. J Biol Chem: 282, 26832–26844. 10.1074/jbc.M702640200 17646164

[17] Samuel F, Hynds DL (2010) RHO GTPase signaling for axon extension: is prenylation important? Mol Neurobiol: 42, 133–142. 10.1007/s12035-010-8144-2 20878268

[18] Spindler SR, Li R, Dhahbi JM, Yamakawa A, Mote P, et al.. (2012) Statin treatment increases lifespan and improves cardiac health in Drosophila by decreasing specific protein prenylation. PLoS One: 7, e39581. 10.1371/journal.pone.0039581 PMC3380867 22737247

[19] Van Doren M, Broihier HT, Moore LA, Lehmann R (1998) HMG-CoA reductase guides migrating primordial germ cells. Nature: 396, 466–469. 10.1038/24871 9853754

[20] Spradling AC, Stern D, Beaton A, Rhem EJ, Laverty T, et al.. (1999) The Berkeley Drosophila Genome Project gene disruption project: Single P-element insertions mutating 25% of vital Drosophila genes. Genetics: 153, 135–177. 10.1093/genetics/153.1.135 PMC1460730 10471706

[21] Strutt DI, Weber U, Mlodzik M (1997) The role of RhoA in tissue polarity and Frizzled signalling. Nature: 387, 292–295. 10.1038/387292a0 9153394

[22] Lee T, Winter C, Marticke SS, Lee A, Luo L (2000) Essential roles of Drosophila RhoA in the regulation of neuroblast proliferation and dendritic but not axonal morphogenesis. Neuron: 25, 307–316. 10.1016/s0896-6273(00)80896-x 10719887

[23] Carlson CA, Kim KH (1973) Regulation of hepatic acetyl coenzyme A carboxylase by phosphorylation and dephosphorylation. J Biol Chem: 248, 378–380. 4692841

[24] Steinberg GR, Kemp BE (2009) AMPK in Health and Disease. Physiol Rev: 89, 1025–1078. 10.1152/physrev.00011.2008 19584320

[25] Zhang FL, Casey PJ (1996) Protein prenylation: molecular mechanisms and functional consequences. Annu Rev Biochem: 65, 241–269. 10.1146/annurev.bi.65.070196.001325 8811180

[26] Lowery LA, Van Vactor D (2009) The trip of the tip: understanding the growth cone machinery. Nat Rev Mol Cell Biol: 10, 332–343. 10.1038/nrm2679 PMC2714171 19373241

[27] Nikolic M (2002) The role of Rho GTPases and associated kinases in regulating neurite outgrowth. Int J Biochem Cell Biol: 34, 731–745. 10.1016/s1357-2725(01)00167-4 11950591

[28] Woo S, Gomez TM (2006) Rac1 and RhoA promote neurite outgrowth through formation and stabilization of growth cone point contacts. J Neurosci: 26, 1418–1428. 10.1523/JNEUROSCI.4209-05.2006 PMC6675502 16452665

[29] Tschape JA, Bettencourt da Cruz A, Kretzschmar D (2003) Progressive neurodegeneration in Drosophila: a model system. J Neural Transm Suppl, 51–62. 10.1007/978-3-7091-0643-3_3 12946048

[30] Cole SL, Vassar R (2006) Isoprenoids and Alzheimer’s disease: a complex relationship. Neurobiol Dis: 22, 209–222. 10.1016/j.nbd.2005.11.007 16406223

[31] Novelli G, D’Apice MR (2012) Protein farnesylation and disease. J Inherit Metab Dis. 10.1007/s10545-011-9445-y 22307208

[32] Brand AH, Perrimon N (1993) Targeted gene expression as a means of altering cell fates and generating dominant phenotypes. Development (Cambridge, England): 118, 401–415. 10.1242/dev.118.2.401 8223268

[33] Kretzschmar D, Tschape J, Bettencourt Da Cruz A, Asan E, Poeck B, et al.. (2005) Glial and neuronal expression of polyglutamine proteins induce behavioral changes and aggregate formation in Drosophila. Glia: 49, 59–72. 10.1002/glia.20098 15390099

[34] Kretzschmar D, Brunner A, Wiersdorff V, Pflugfelder GO, Heisenberg M, et al.. (1992) Giant lens, a gene involved in cell determination and axon guidance in the visual system of Drosophila melanogaster. Embo J: 11, 2531–2539. 10.1002/j.1460-2075.1992.tb05318.x PMC556728 1628618

[35] Benzer S (1967) Behavioral mutants of Drosophila isolated by countercurrent distribution. Proc Natl Acad Sci USA 58, 1112–1119. 10.1073/pnas.58.3.1112 PMC335755 16578662

[36] Strauss R, Heisenberg M (1993) A higher control center of locomotor behavior in the Drosophila brain. J Neurosci: 13, 1852–1861. 10.1523/JNEUROSCI.13-05-01852.1993 PMC6576564 8478679

[37] Swanson TL, Knittel LM, Coate TM, Farley SM, Snyder MA, et al.. (2005) The insect homologue of the amyloid precursor protein interacts with the heterotrimeric G protein Go alpha in an identified population of migratory neurons. Dev Biol: 288, 160–178. 10.1016/j.ydbio.2005.09.029 PMC2862231 16229831

[38] Orgad S, Dudai Y, Cohen P (1987) The protein phosphatases of Drosophila melanogaster and their inhibitors. Eur J Biochem: 164, 31–38. 10.1111/j.1432-1033.1987.tb10988.x 3030753

[39] Bradford MM (1976) A rapid and sensitive method for the quantitation of microgram quantities of protein utilizing the principle of protein-dye binding. Anal Biochem: 72, 248–254. 10.1016/0003-2697(76)90527-3 942051

